# How Does Information Overload Affect Consumers’ Online Decision Process? An Event-Related Potentials Study

**DOI:** 10.3389/fnins.2021.695852

**Published:** 2021-10-21

**Authors:** Minjing Peng, Zhicheng Xu, Haiyang Huang

**Affiliations:** School of Economics and Management, Wuyi University, Jiangmen, China

**Keywords:** information overload, purchase decision, P2, P3, LPC, time-varying analysis

## Abstract

One of the advantages of e-retailers is their capability to provide a large amount of information to consumers. However, when the amount of information exceeds consumers’ information processing capacities, it will lead to worse decision quality and experience, causing the information overload effect. In this study, the event-related potentials (ERPs) were applied to examine the hidden neural mechanism of the impact of information overload on consumers’ decision processes. Behavioral data showed that people would spend more time making decisions when faced with information overload. Neurophysiologically, consumers would invest less attentional resources in the high amount of information (HAI) condition than those in the low amount of information (LAI) condition and lead to less positive P2 amplitudes. The HAI condition would increase decision difficulty than would the LAI condition and result in smaller P3 amplitudes. In addition, an increased late positive component (LPC) was observed for the HAI condition in contrast to the LAI condition, indicating that consumers were more inclined to have decision process regret when consumers were overloaded. We further investigated the dynamic information processing when consumers got over information overload by mining the brain’s time-varying networks. The results revealed that during the decision process and the neural response stage, the central area controlled other brain regions’ activities for the HAI condition, suggesting that people may still consider and compare other important information after the decision process when faced with information overload. In general, this study may provide neural evidence of how information overload affects consumers’ decision processes and ultimately damages decision quality.

## Introduction

With the rapid development of information technology, the way of shopping has undergone enormous changes. According to the National Bureau of Statistics, online retail sales in China totaled $1.8 trillion for 2020. Online shopping has become the essential impetus for the growth of consumption. Retailers can provide consumers with numerous products and a mass of product information in the online environment because of the infinite shelf space (Chen et al., 2009). Therefore, it is relevant to explore consumer behavior in this new environment.

Previous research has demonstrated that more information might play a crucial role in improving the quality of decision making (Berger et al., 2007; Scheibehenne et al., 2010). The most significant advantage of the internet environment is reducing the time that consumers spent searching for information, enabling them to make informed purchase decisions. While reducing information asymmetry, people are gradually surrounded by the information overload problem. The information overload effect states that when the input exceeds the processing capacity, more negative effects will ensue (Eppler and Mengis, 2004; Roetzel, 2018). In terms of decision behavior, information overload can occupy numerous cognitive resources (Nagar and Gandotra, 2016) and damage decision quality (Jacoby, 1984; Sasaki et al., 2011). In addition, the large amounts of information will further aggravate the decision difficulty (Sicilia and Ruiz, 2010; Hu and Krishen, 2019), resulting in decision delay and more inefficiency (Iyengar and Lepper, 2000). In the era of the experience economy, the main focus of marketing discussion is how to improve the consumer buying experience (Chen et al., 2009). Previous studies showed that as the amount of information increased, consumers would feel more negative emotions, less decision satisfaction (Lee and Lee, 2004), and more buyer’s remorse (Inbar et al., 2011; Chernev et al., 2015). In summary, previous studies about the net impact of the amount of information on consumer decisions were contradictory. Moreover, most studies focused only on consumer decisions without an in-depth investigation of the cognitive and neural basis involved in consumers’ decision process.

Unlike most previous research that used self-reported approaches, the current study used event-related potentials (ERPs), which is characterized by its great time resolution, contributing to uncovering the time course of brain activity, to explore the underlying neural mechanism of how information overload affects consumers’ decision process in the online environment (Luck et al., 2000). The main weakness of self-report is that it does not open the brain’s black box and mine the related information processing activities (Wang et al., 2016). Furthermore, psychometric self-reported data are often blamed for causing subjective bias, which hinders objective measurements (Kuan et al., 2014). During the experiment, participants were asked to make buying decisions under different online information environments. According to prior studies on purchase decisions, the P2, P3, and late positive component (LPC) are of interest to this study. To discover the mechanism of information overload, we used time-varying network analysis to reveal the dynamic network structures under different information load conditions.

The amplitudes of P2 are related to the attentional resources invested by the participants (Huang and Luo, 2006). It is an early positive component with a peak latency of about 200 ms after stimulus onset and mainly distributes over the frontal and central areas (Carretié et al., 2001). Previous studies suggested that larger amplitudes would be found in the negative stimuli than the positive ones (Carretié et al., 2001; Jing et al., 2019). In contrast, some studies concluded that P2 amplitudes were small when the potency of stimulus material was negative (Yuan et al., 2007). However, the similarity of the two types of studies is that they agree that larger P2 amplitudes were observed when more attentional resources were invested (Mercado et al., 2006). According to cognitive miser theory (Taylor and Fiske, 1978; Evans and Stanovich, 2013), people will be more inclined to adopt decision strategies that save attentional resources in the high amount of information (HAI) condition. Therefore, we hypothesize that when faced with information overload, consumers will allocate less attentional resources, which can be reflected in the smaller P2 amplitudes.

Besides P2, P3 also can represent attentional resources allocation (Polich, 1987). It is a positive component with a frontal–parietal scalp distribution peaking at about 300–400 ms (Folstein et al., 2008; Ma et al., 2008). Related research on ERPs showed that the more attention resources that people allocated to stimulus materials, the larger P3 amplitudes were evoked (Kok, 2001). In addition, it is closely related to information processing in the brain. Prior studies indicated that the amplitudes of P3 were affected by the decision difficulty and confidence, with the more significant difficulties or lacking confidence eliciting the less positive P3 amplitudes (Nieuwenhuis et al., 2005; Qin and Han, 2009). In the present study, we predict information overload will increase the decision difficulty and reduce decision confidence and then the reduced P3 will be observed.

LPC reflects emotional valence and arousal (Amrhein et al., 2004; Cuthbert et al., 2000). It commonly peaks approximately 500–800 ms and is generally found in the frontal and central sites (Todd et al., 2008). Previous studies indicated that negative stimuli could elicit larger LPC amplitudes (Schupp et al., 2004; Strauss et al., 2018). In emotional arousal terms, past works consistently suggested that the enhanced LPC amplitudes could be observed in high arousal emotions compared with low arousal emotions. Pollatos et al. (2005) showed that people with the better introspective experience of their physiological arousal had higher arousal ratings, evidenced by the more positive LPC amplitudes. In our study, the HAI condition may cause a more cursory process than the LAI condition, producing a sense of haste in evaluating alternatives and a strong negative emotional response (i.e., remorse) under time pressure. Thus, we speculate that information overload will lead to heightened remorse arousal, which can be reflected in the larger amplitudes of LPC.

Notably, the decision involves multiple brain regions and includes the interactions among these brain regions (Petersen and Sporns, 2015). Given the millisecond information processing capacity of the brain, ERPs methods may be more advantageous in constructing time-varying network patterns of distinct cognitions (i.e., attention, decision, and motor imagery) than functional magnetic resonance imaging (fMRI) measures. Li et al. (2016) suggested that the brain corresponded to different network modes in various decision stages by constructing time-varying networks. Si et al. (2018) revealed the distinct network structures when people accepted or refused unfair proposals by the time-varying network analysis method. In the current study, people may adopt diverse information processes modes under different information load conditions. As a result, we hypothesize that there can be various dynamic network patterns between the LAI and HAI conditions.

Taken together, although it was not clear how information overload affected the mental processes of consumers, previous research conclusions resulted in the following expectations: (1) the attenuated P2 and P3 components and an increased LPC could be elicited when consumers faced information overload. (2) The different information environments would involve diverse time-varying network structures.

## Materials and Methods

### Participants

We recruited 40 participants (20 males, mean age = 23.5 years, *SD* = 1.26) with right-handedness from the School of Economics & Management at Wuyi University. All participants reported no family history of neurological disease or mental illness and normal or corrected-to-normal vision. The experimental program was approved by the School of Economics & Management at Wuyi University. Participants enrolled in the experiment provided written informed consent at the beginning of the experiment. They were paid for their participation.

### Materials

Fruits were selected as the category for our experiment. Provenance is the most recognized brand label for Chinese agricultural products (Lin, 2020). It is well-known that meteorological factors, such as temperature, precipitation, and sunshine, will affect the growth of agriculture. In addition, climatic conditions also might shape agricultural production (Holzkämper et al., 2013). Thus, besides the brand and price information, we also provided the climate, temperature, precipitation, and sunshine suitability of the origin to characterize the fruit quality.

Two versions of the stimuli were designed by manipulating the amount of information. Estimates of the optimal amount of information to be processed by an individual have ranged from 4 to 10 (Sicilia and Ruiz, 2010). When the volume of information exceeds 10, the individual information processing ability will decrease. The final amount of information under each condition (6 and 12 for the LAI and HAI conditions, respectively) was determined through a pretest. Beyond two images of the same kind and similar shape, each picture includes a corresponding amount of information condition. To avoid some heuristic clues, we provided fictitious brands, comparable prices, and neutral ratings (i.e., three-star ratings and four-star ratings). We processed all pictures by Photoshop software to ensure consistency in the background, position, brightness, and color and resized them to a uniform size (810 × 410 pixels).

### Procedure

**FIGURE 1:**
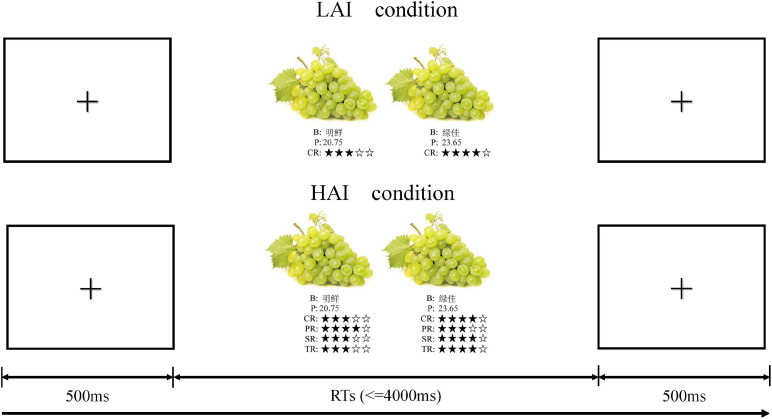
Experimental task. Participants were instructed to make purchase decisions under different information volume conditions. B for brand, P for price, CR for climate rating, PR for precipitation rating, SR for sunshine rating, and TR for temperature rating.

Participants sat in a comfortable chair located in a soundproofed room. They were required to avoid frequent blinking and body movement as possible. Before the formal experiment started, they were instructed to read the guideline of the investigation and visited a simulated fresh products e-commerce platform, imagining that they were choosing fruits online. The experiment consisted of two blocks, each containing 100 trails. During the experiment, the participants were instructed to perform 100 LAI tasks and 100 HAI tasks. The E-prime 3.0 software was used to control the stimuli and collect data. As shown in Figure 1, each trial began with a fixation of “+” presented for 500 ms on the white screen, which was followed by decision tasks to be performed. These decision tasks can be either a LAI task or a HAI task, randomly assigned by the program so that participants cannot predict the type of the next task. During each decision task, half of the participants were instructed to press “A” for “choose the left one” and “B” for “choose the right one,” and the others had the opposite pattern. They had a maximum of 4,000 ms to make their decision by pressing the key. Four practice trials were conducted for each participant before the start of the formal experiment.

### Electroencephalogram Recording

The electroencephalogram (EEG) was recorded (bandpass 0.01–100 Hz, sampling rate 500 Hz) by Brain Products Amplifier, using an electrode cap with 32 Ag/AgCl electrodes mounted according to the 10–20 system. The linked mastoid served as an online reference. The electrode impedance was kept below 10 KΩ during the whole experiment.

### Data Analysis

**FIGURE 2:**
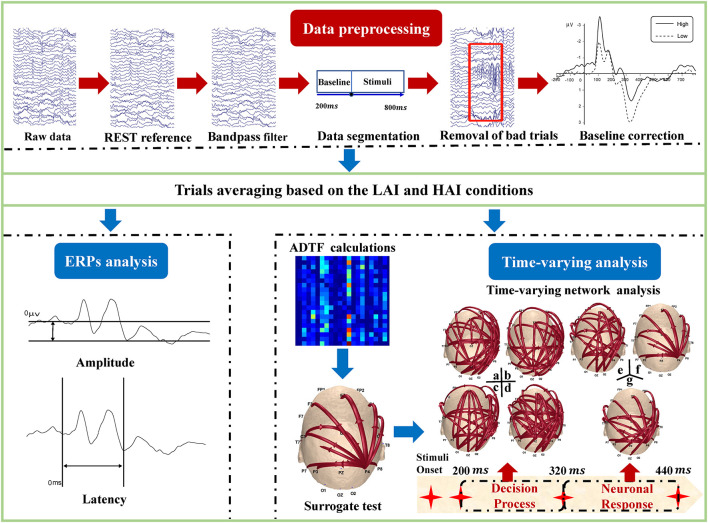
Electroencephalogram (EEG) data analysis procedure. EEG data preprocessing, event-related potentials (ERPs) analysis, and time-varying analysis were involved in the analysis protocol.

The analytical programs were shown in Figure 2, including preprocessing and time-varying network construction and analysis. The details of the sub-procedures can be described as follows.

#### Electroencephalogram Data Preprocessing

EEGLAB 13.4.4 software was used to process the offline EEG data. The data were preprocessed with procedures consisting of Reference Electrode Standardization Technique (Yao, 2001) referencing, 0.1–30 Hz bandpass filtering, [–200 ms, 800 ms] data segmenting, and [−200 ms, 0 ms] baseline correcting and artifacts removal (±75 μ V as the threshold). The EEG epochs were then averaged for the two conditions.

#### Time-Varying Network Analysis

To minimize the effect of volume conduction, 20 canonical electrodes of the international 10–20 system were selected (Si et al., 2018). After preprocessing, these artifact-free trials that were applied to construct the time-varying network were down-sampled to 25 Hz for each participant (i.e., there is a 40-ms interval between two adjacent sampling points). To obtain reliable time-varying network results, all of these networks corresponding to individual trials were averaged across all trials for the different tasks.

#### Time-Varying Multivariate Adaptive Autoregressive Model

For each HAI/LAI condition trial time series, the following formula (1) is used to calculate the time-varying multivariate adaptive autoregressive (tv-MVAAR) model,


(1) $$\text{X}\left(t\right)=\sum_{i=1}^{p}\text{A}\left(i,t\right)\text{X}\left(\text{t}-\text{i}\right)+\text{E}\left(t\right)$$

where X(t) is the EEG data vector over the whole-time window of [−200 ms, 800 ms], A(i, t) is the tv-MVAAR model coefficient matrix that was estimated by the Kalman filter algorithm (Arnold et al., 1998), E(t) is the Gaussian white noise, and p is the optimal model sequence, determined by the Akaike information criterion (AIC).

#### Adaptive Directed Transfer Function

H(f, t) can be calculated by transforming the frequency domain of A(i, t) from the tv-MVAAR model coefficients. The element H_ij_(f, t) in H(f, t) implies the flow of information from the jth to ith node at time point t and frequency f.


(2) A(f,t)X(f,t)=E(f,t)

(3) $$\text{X}\left(f,t\right)={\text{A}}^{-1}\left(f,t\right)\text{E}\left(f,t\right)=\text{H}\left(f,t\right)\text{E}\left(f,t\right)$$

where H(f, t) = A^−1^(f, t), $A\left(f,t\right)=\sum_{\text{k}=0}^{\text{p}}{\text{A}}_{\text{k}}\left(t\right){\text{e}}^{-\mathrm{j2}\pi \text{f}\Delta \text{tk}}$, A_k_ is the matrix of the model coefficients, and X(f, t) andE(f, t) are the transformations of the signal X(t) and its corresponding white noise E(t) in the frequency domain, respectively.

The normalized Adaptive Directed Transfer Function (ADTF) represents the flow of directed information from the jth to ith node, which is generally defined between (0, 1) as


(4) $$\gamma_{\mathrm{ij}}^{2}\left(f,t\right)=\frac{{\left|{\text{H}}_{\mathrm{ij}}\left(f,t\right)\right|}^{2}}{\sum_{m=1}^{n}{\left|{\text{H}}_{\mathrm{im}}\left(f,t\right)\right|}^{2}}$$

Finally, the composite ADTF is solved by the mean ADTF values over the frequency band of interest [f_1_,f_2_] as


(5) $$\Theta_{\mathrm{ij}}^{2}\left(t\right)=\frac{\sum_{k=f_{1}}^{f_{2}}{\text{r}}_{\mathrm{ij}}^{2}\left(k,t\right)}{{\text{f}}_{2}-{\text{f}}_{1}}$$

More detailed information regarding the ADTF method can be found in Wilke et al. (2008). Given the frequency band for P300, [1 *Hz*, 10 Hz] was selected as the range of the average ADTF values for the oriented information flow.

## Results

### Manipulation Check of the Pretest

An online questionnaire that 201 participants (95 males and 106 females; mean age = 23.58 years) completed was used to check the manipulation for the two levels of amount of information. In this pretest, the perceived amount of information levels was measured with one 5-point Likert-scale question adapted from Lee and Lee (2004): “There were many characteristics of fruits to consider.” The mean scores for the LAI and HAI conditions were 2.90 and 3.85, respectively. Statistical tests were conducted by SPSS 25.0 software. Moreover, the independent-samples *t*-test showed that the difference was significant [*t*(199) = −15.50, *p* < 0.001], which implied that the manipulation of the amount of information would change the participants’ perception of the amount of information.

### Behavioral Data

Decision response times (RTs) indicated the periods from the moment the stimuli began to present to the effective decision was made. It means the time that one requires for making the purchase decision. The result showed that RTs in the HAI condition (mean = 1511.69 ms, *SD* = 498.94, and median = 1390 ms) were more prolonged than those in the LAI condition (mean = 1106.68 ms, *SD* = 408.81, and median = 950 ms). Wilcoxon signed-rank test showed a significant difference between HAI and LAI conditions (Z = −43.97, *p* < 0.001).

### Event-Related Potentials Data

**FIGURE 3:**
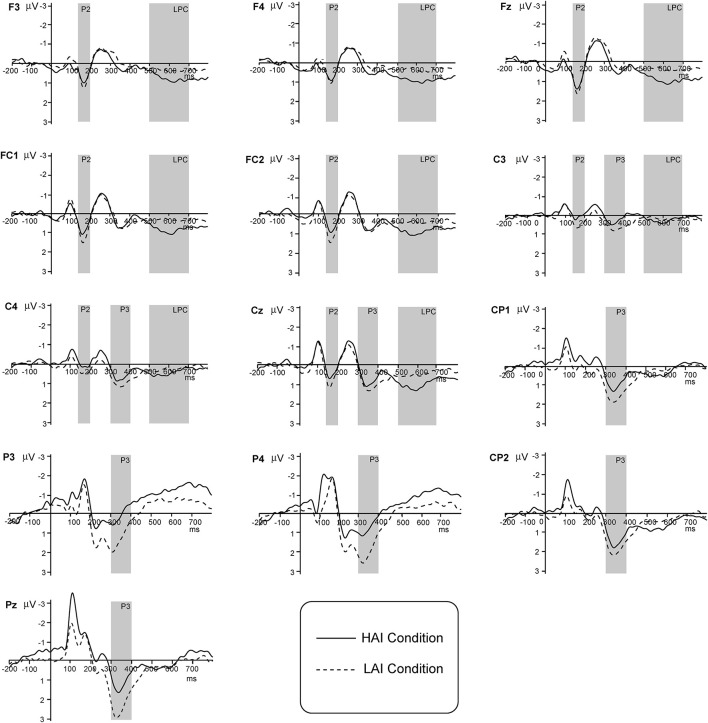
Grand averaged event-related potentials (ERPs) of P2, P3, and late positive component (LPC) elicited by different amounts of information condition (high vs. low).

The grand-averaged ERPs waveforms for two amounts of information conditions are shown in Figure 3. As might be expected, P2, P3, and LPC were successfully elicited in our results. Based on relevant research mentioned in the *Introduction*, eight electrodes (F3, F4, FC1, FC2, Fz, C3, C4, and Cz) from the frontal to central regions were selected for P2 and LPC analysis, and eight electrodes (C3, C4, CP1, CP2, Cz, P3, P4, and Pz) in the central–parietal areas were selected for P3 analysis. The ERPs amplitudes of time windows of 140–200, 300–400, and 500–700 ms were averaged for P2, P3, and LPC analysis, respectively. A 2 (amount of information) × 8 (electrode) within-subjects repeated-measures ANOVA was conducted for the P2, P3, and LPC. The Greenhouse–Geisser correction (Greenhouse and Geisser, 1959) was applied when necessary.

For the P2 component, the repeated-measures ANOVA results indicated that the main effect of the amount of information was significant [$F\left(1,39\right)=7.215,p=0.011<0.05,\eta_{P}^{2}=0.156$]. The main effect of the electrode sites was also significant [$F\left(7,273\right)=7.703,p<0.001,\eta_{P}^{2}=0.165$]. However, the interaction effect of these two factors was not significant [$F\left(7,273\right)=0.844,p=0.491,\eta_{P}^{2}=0.021$]. In addition, the P2 amplitudes elicited by the LAI condition (mean = 2.034 μ V, *SD* = 1.78) were more positive than those elicited by the HAI condition (mean = 1.606 μ V, *SD* = 1.56) significantly.

The analysis of P3 average amplitudes showed that a significant main effect of the amount of information [$F\left(1,39\right)=12.862,p<0.001,\eta_{P}^{2}=0.248$]. Also, the main effect of the electrode sites was significant [$F\left(7,273\right)=15.010,p<0.001,\eta_{P}^{2}=0.278$], and their interaction effect was significant [$F\left(7,273\right)=7.580,p<0.001,\eta_{P}^{2}=0.163$]. *Post-hoc* comparisons indicated that the P3 amplitudes elicited by the LAI condition were significantly larger than those elicited by the HAI condition at the points of C3, CP1, CP2, P3, P4, and Pz. However, there was no significant difference at other points (*p* > 0.05).

As for LPC, the ANOVA results indicated that the main effects of the amount of information [$F\left(1,39\right)=9.348,p=0.004<0.05,\eta_{P}^{2}=0.193$] and the electrode [$F\left(7,273\right)=12.574,p<0.001,\eta_{P}^{2}=0.244$] were significant, and also their interaction effect was significant [$F\left(7,273\right)=2.554,p=0.044<0.05,\eta_{P}^{2}=0.061$]. *Post-hoc* comparisons showed that the LPC amplitudes evoked by the LAI condition were significantly larger than those evoked by the HAI condition at the points of F3, F4, FC1, FC2, Fz, and Cz. However, there was no significant difference at other points (*p* > 0.05).

### Time-Varying Network

**FIGURE 4:**
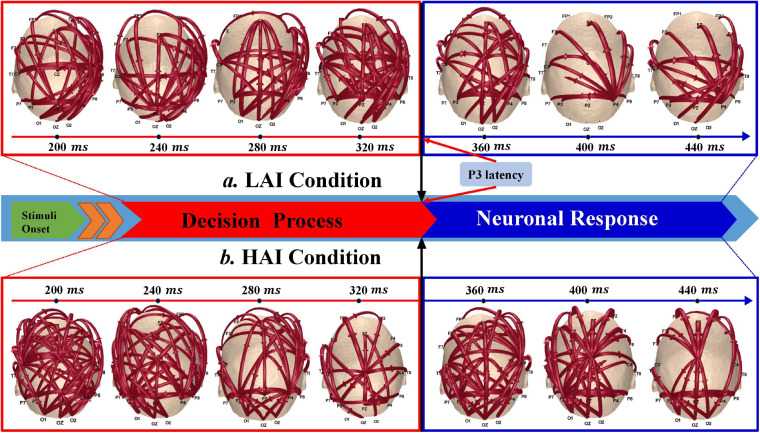
Dynamic changes of information flow for the low amount of information (LAI) and high amount of information (HAI) conditions. Brown lines represent the information flow between two electrodes, and arrows indicate the direction of information flow. **(a)** Low amount of information condition. **(b)** High amount of information condition.

The P3 is induced by the response of neurons to external stimuli after the decision-making process stage (Picton, 1992). Moreover, the P3 peak for electrode Cz occurred at approximately 369.60 ± 45.97ms. Therefore, based on the P300-related research (Wang et al., 2014; Li et al., 2016) and the P3 waveform, we distinguished the corresponding time-varying networks into two substages, i.e., decision process (200–320 ms) and neuronal response stage (320–440ms). The current results revealed that the source region switched from the center area (CA) to the right posterior parietal cortex (rPPC) in the LAI condition, corresponding to the decision process and neural response stage, respectively (Figure 4). During the decision process stage, the CA controlled different brain regions by giving commands. However, the rPPC then served as a new source to control activities in other brain regions. In contrast, the CA held brain regions by issuing instructions during the decision process and the neural response stages in the HAI condition.

## Discussion

With the use of the ERPs method, the present study explored how information overload affected consumers’ decision process in the online platform. In this experiment, participants were asked to make buying decisions under different online information environments. Results showed that longer RTs, smaller P2 and P3, and a larger LPC were observed when consumers were overloaded. Furthermore, we used the approach of time-varying network analysis to mine distinct dynamic network modes corresponding to various decision-making phases when individuals experienced information overload.

Previous research indicated that RTs were associated with decision difficulty and cognitive load (Sweller, 1988; Wang et al., 2016; Shen et al., 2018). In a study of Shen et al. (2018), they asked consumers to make purchase decisions under different review star rating conditions and found that the three-star ratings led to increased RTs, indicating that neutral review star ratings made purchase decisions more difficult. In the current study, when the amount of information increases, the number of alternatives increases and makes decisions more difficult. Therefore, longer RTs were found in the HAI condition than those in the LAI condition.

ERPs results reflected the cognitive and neural processes of product evaluation and buying decisions. The P2 components are positively related to attention resources (Mercado et al., 2006; Jing et al., 2019). In addition, P3 amplitudes are inversely proportional to decision difficulty (Qin and Han, 2009). It is consistent with a study of Nieuwenhuis et al. (2005), which concluded that the smaller P3 amplitudes were evoked when consumers lack confidence in the decision. In our study, when the amount of information on e-commerce platforms is overloading, information-processing cost increases. Consistent with minimizing cognitive cost according to the minimax principle, consumers will shift to a more cost-saving non-compensatory strategy. Therefore, fewer attention resources will be devoted to the decision when people are overloaded. On the contrary, when faced with the LAI condition, consumers have confidence in making decisions. Thus, they will devote more attention resources to a comparative analysis by compensatory rule. Therefore, the P2 and P3 in the LAI condition were larger than those in the HAI condition.

The LPC is sensitive to mood changes. Previous studies had shown that the larger LPC represented high emotional arousal (Schupp et al., 2004; Huang and Luo, 2006). Furthermore, it also reflects higher-level cognitive processes, such as the elaborative processing of decision information (Hajcak and Nieuwenhuis, 2006; Meule et al., 2013; Shang et al., 2020). In a study of Meule et al. (2013), they asked consumers to make food choices under different cognitive strategies for suppressing food craving and found that the LPC amplitudes would be increased when participants considered the long-term consequences of eating high-calorie foods. Likewise, Shang et al. (2020) suggested that when consumers considered hedonic products were unnecessary as compared with utilitarian products, an enlarged LPC amplitude could be found. Extending the above insights to our study, information overload will lead to numerous tradeoffs and comparisons. Thereby, people will feel uncertain about whether their forthcoming decision is the best one. In this context, emotional arousal might arise from counterfactual thinking between the outcome of the chosen option and the unchosen option. Therefore, more positive LPC amplitudes were evoked by the HAI condition than those evoked by the LAI condition. In addition, the differences in LPC were mainly found in the prefrontal cortex. This result is consistent with previous regret studies that used the fMRI method, which suggested that the orbitofrontal cortex (OFC) was involved in regret processing (Coricelli et al., 2005). In sum, we predict the LPC is likely the product of emotional arousal (i.e., regret) in which the brain’s emotional evaluation of the anticipated results causes a higher cognitive evaluation (i.e., counterfactual thinking).

Based on the ADTF, we further investigated dynamic cognitive processing by mining its time-varying networks. The representative cognitive processes consist of three distinct phases: information integration, decision-making processes, and neuronal responses (Fellows, 2004). P3 component is elicited by the neuronal response to an external stimulus following the decision process stage (Picton, 1992). These constructed time-varying networks determined different network patterns corresponding to the decision process and neuronal response phases of P3. Inspired by prior works, we hypothesize that the need for various brain functions in distinct stages of information processing resulted in a shift in activation regions (Si et al., 2018).

Previous studies had shown that the decision phase typically occurs after the information integration stage (Gong et al., 2012). There is a certain extent of overlap between these two stages, which was also reflected in our results. As can be seen in Figure 4, for the LAI and HAI conditions, the networks in the early decision process stage (e.g., 200- and 240-ms time points) showed similar network structures with apparent information interactions observed among multiple regions, such as the frontal cortex and occipital lobe, which represent information integration. However, the information interactions were denser for the HAI condition compared with the LAI condition. One reason might be that more information will need to be processed for the HAI condition. Figure 4a further demonstrates that the CA served as a core source to control relevant brain activities during the following decision process stage. Except for the role in the motor process, these regions are closely related to mental functions such as thinking and planning (Fan et al., 2005; Neubert et al., 2015). Therefore, the CA controls the cognitive process in the decision process phase, involving information integration, decision making, and response preparation.

Then, Figure 4 indicates distinct brain network patterns during the neuronal response stage for the HAI and LAI conditions. Specifically, the source areas switched from the CA to the rPPC in the LAI condition, corresponding to the decision process and neural response stage, respectively (see Figure 4). In our study, participants were instructed to evaluate product information, which was strongly related to working memory and attention. Previous research reported that the rPPC was closely linked to cognitive and attentional control (Malhotra et al., 2009; Shomstein, 2012; Zhong et al., 2019). Thus, the rPPC served as the new core source to regulate other brain regions. In addition, the time-varying networks in Figure 4a also suggested that the bottom-up network couplings from the rPPC to the prefrontal cortex was the primary pattern during the neuronal response stage. The related research had shown the importance of these information flows between the frontal and parietal cortices for reasoning and decision (Daffner et al., 2003; Fan et al., 2005). Furthermore, these brain regions are highly correlated with cognitive processes, such as attention and work memory. Therefore, these results can explain the importance of these information flows between the rPPC and the frontal cortex to purchase decisions. However, there was no apparent shift in the core nodes for the HAI condition (see Figure 4b). We speculate that one likely reason is that remorse would promote people to improve the rationality of decision making. When anticipated regret is aroused, it might prompt people to increase information gathering or adapt decision strategies for preventing hasty decisions (Zeelenberg et al., 2002). Therefore, the CA, which is closely associated with thinking and planning, still serves as the focal source controlling other brain areas when consumers faced a large amount of information.

### Limitations

This study preliminarily suggested the existence of the information overload effect from a neuroscience perspective. However, there are some limitations of this study that have to be acknowledged. Given that the mechanisms that the impact of the amount of product and attribute quantities on information overload may be different, we manipulated the amount of information only through the number of attributes for each product, which seems to be a typical way of adding information to e-commerce platform (Lee and Lee, 2004; Sicilia and Ruiz, 2010). In addition, only two information load groups (high and low) were considered, and six product attributes were designed for the HAI condition in this study. Consumers might be easier to evaluate product information and make decisions when faced with fewer options. Furthermore, we have balanced gender and tested whether there was a significant interaction with the amount of information in our study. While we did not observe any significant effects of gender (*p* > 0.05), the selectivity hypothesis suggested that males and females are distinct information processing patterns (Meyers-Levy and Sternthal, 1991). In the field of consumer research, researchers have focused on how gender differences affect consumer behavior (Meyers-Levy and Loken, 2015; Papyrina, 2019). Thereby, the gender factor may play an interesting role in how information overload affects consumers’ online decision process. Future studies might consider more deeply investigating this possibility. Finally, the findings of this study only illustrated that people would devote less cognitive resources to making decisions, relying on heuristic cues. However, we failed to reveal which heuristic cues are of interest to users. This critical information can be used to simplify product information and improve the quality of information, which can help companies increase competitiveness. Therefore, future research can investigate which information is important for consumers when faced with information overload by using the eye movement technique.

## Conclusion

In sum, this study explored how information overload influenced consumers’ decision process and its hidden neural basis. The current findings indicated that information overload might damage the decision process, reflected in decreased P2 and P3 amplitudes and an increased LPC when people faced information overload. We suggested that P2 reflected automatic attentional resources allocation, P3 represented decision difficulty and confidence, and LPC could be regarded as decision process regret arousal. Furthermore, we also used time-varying network analysis to mine the different network modes corresponding to the various decision stages. In the LAI condition, the CA served as the core source during the decision process phase. The source area was switched to the rPPC during the neuronal response stage, suggesting different decision stages may trigger different network structures. Interestingly, in the decision process and neuronal response stages, the CA controlled other brain regions under the HAI condition, which indicated that consumers might consider and compare additional information after the decision process stage when faced with information overload. In general, the present study revealed the neural mechanism of the information overload effect, which would be helpful for future marketing research.

## Data Availability Statement

The datasets presented in this study can be found in online repositories. The names of the repository/repositories and accession number(s) can be found here: https://osf.io/2pe93/.

## Ethics Statement

The studies involving human participants were reviewed and approved by the School of Economics & Management, Wuyi University. The patients/participants provided their written informed consent to participate in this study.

## Author Contributions

ZX, HH, and MP conceived and designed the experiments. ZX and MP performed the experiments. ZX analyzed the data. ZX and HH wrote and edited the manuscript. All authors contributed to the article and approved the submitted version.

## Conflict of Interest

The authors declare that the research was conducted in the absence of any commercial or financial relationships that could be construed as a potential conflict of interest.

## Publisher’s Note

All claims expressed in this article are solely those of the authors and do not necessarily represent those of their affiliated organizations, or those of the publisher, the editors and the reviewers. Any product that may be evaluated in this article, or claim that may be made by its manufacturer, is not guaranteed or endorsed by the publisher.
